# Components and Application Plans for Designing a Korean Forest Therapy Prescription Model: Using Case Examination and a Focus Group Interview (FGI)

**DOI:** 10.3390/healthcare13080866

**Published:** 2025-04-10

**Authors:** Pyeongsik Yeon, Neeeun Lee, Sinae Kang, Gayeon Kim, Youngeun Seo, Sooil Park, Kyungsook Paek, Saeyeon Choi, Seyeon Park, Hyoju Choi, Gyeongmin Min, Jeonghee Lee

**Affiliations:** 1 Department of Forest Sciences, Chungbuk National University, Cheongju 28644, Republic of Korea; well@chungbuk.ac.kr (P.Y.); hin0358@chungbuk.ac.kr (H.C.); 2 Graduate Department of Forest Therapy, Chungbuk National University, Cheongju 28644, Republic of Korea; share1227@chungbuk.ac.kr (N.L.); sinae375@chungbuk.ac.kr (S.K.); rkdus6520@naver.com (G.K.); youngeun3311@chungbuk.ac.kr (Y.S.); wce5644@chungbuk.ac.kr (S.P.); ksbaek75@chungbuk.ac.kr (K.P.); snoopy@chungbuk.ac.kr (S.C.); 0291psy@chungbuk.ac.kr (S.P.); 3 Forest Human Service Division, Future Forest Strategy Department, National Institute of Forest Science, Seoul 02455, Republic of Korea

**Keywords:** forest therapy, forest healing, nature-based therapy, forest therapy prescription, nature-based prescription model, focus group interview (FGI)

## Abstract

**Background:** Although forest therapy services in South Korea have demonstrated mental and physical effects, there is no established system for forest therapy prescriptions. To this end, it is necessary to devise a systematic model for the introduction of forest therapy prescriptions by linking the existing forest therapy infrastructure and medical services. Therefore, this study aimed to derive the components and application plans needed to devise a forest therapy prescription model for the spread of medical-linked forest therapy services and to secure a forest therapy prescription infrastructure. **Methods:** To this end, Korean and foreign cases of prescription models and healthcare service provision systems were analyzed to derive the necessary components for prescription models. Subsequently, a Focus Group Interview (FGI) was conducted with eight experts in the fields of forest therapy and welfare, psychiatry, and health and nursing, and opinions were derived regarding the conception and empirical application of the forest therapy prescription model through content analysis. **Results:** As a result of the study, five components (clear role-sharing and a collaboration system, a continuous system, customized service provision, various technologies and content, and a database-based prescription system) were derived from cases of prescription models and healthcare service provision systems according to field. Furthermore, the FGI identified three primary topics: stakeholders’ scope and role, procedures and effectiveness, and additional considerations. Each was categorized into eight sub-categories relevant to the design of the forest therapy prescription model. **Conclusions:** These results can be used as basic data for devising a systematic Korean forest therapy prescription model in which forest therapy and medical services are linked, providing a foundation for personalized forest therapy prescriptions to be implemented.

## 1. Introduction

Recently, as the trend emphasizing wellness, relaxation, and healing has evolved, there has been an increase in the demand for and interest in healing, health promotion, and clinical effects utilizing natural resources, such as forests, oceans, and rural areas [1]. In particular, exposure to forests and green spaces has been reported to positively affect human stress recovery, as well as physical and mental indicators [2,3,4,5]. Accordingly, in some countries, such as the United States, United Kingdom, and New Zealand, natural prescription systems have been implemented in collaboration with medical institutions to promote health and prevent, manage, and treat diseases. In some countries, specialists in nature prescriptions provide on-site guidance for programs [6], and governments partially subsidize the associated costs [7,8,9,10].

Although there is no standardized definition of natural prescriptions in either the domestic or international context, they are generally described as “programs or interventions aimed at encouraging patients or clients to spend time in nature to improve their health and well-being” [11]. Natural prescription activities include physical, mental, and social activities and may be provided as an unstructured prescription for forest bathing or physical activities or as a structured prescription for a health and well-being program and a natural exploration program [12]. According to Nguye et al. [13], natural prescriptions initially emerged as a potential solution to induce subjects to have more time and more opportunities to make contact with nature than previously, and the effect was found to be greater when health or social experts recommend interventions. Notably, the benefits of natural prescriptions are that they have a lower cost, are safer than conventional drug treatment methods, and can have complex effects such as increased physical activity, increased psychological stability, and increased social connectivity [14].

In Korea, the concept of forest therapy, defined as “immune-strengthening and health-promoting activities that utilize various elements of the forest, including scents and scenic views”, was systematically introduced as a natural-based healing approach in response to the demand of forest visitors seeking health-promoting effects. Since the establishment of the first healing forest as a forest therapy facility in 2010, 76 healing forests have been developed and in operation since December 2023. Furthermore, based on the “Forest Culture and Recreation Act”, forest healing instructors are trained to develop and implement forest therapy programs in natural recreation forests, forest baths, healing forests, and forest paths [15].

Forest-based interventions are provided in various forms, including forest activities, experiences, and structured forest therapy programs. Most effectiveness verification studies were conducted in Asia, including Korea, Japan, and China [16,17]. Stress relief [18], immune function improvement [19], and depression and anxiety level improvements [20] were reported as major effects, indicating physical and mental benefits. In particular, a study of patients with physical symptoms found that forest-based interventions significantly alleviated symptoms in menopausal women [21] and positively affected health promotion and cortisol hormone levels in patients with cancer [22,23]. In addition, in a study of patients with mental symptoms, an improvement in the mental health of patients with emotional and mental disorders [24], an improvement in the cognitive function scores of patients with mild cognitive impairment [25], an increase in the clinical recovery scores of patients with schizophrenia [26], and an improvement in the symptoms of depressed patients [27] were reported. As such, forest-based interventions have the potential to be expanded not only for use in health promotion or stress relief, but also to achieve symptom relief through prescriptions provided by medical personnel.

You et al. [28] explained the forest therapy prescription as “a prescription for health intervention activities through forests and nature through the intervention of medical personnel for the promotion, rehabilitation and recovery of patients”, and said that it can be applied to promote health, improve mental health, and to manage chronic disease, as well as to encourage rehabilitation and recovery. The Korea Forest Service is expanding forest therapy services for diverse populations, including patients with dementia due to aging, and to provide support for pregnancy and childbirth, as well as daily recovery for COVID-19 professionals. Additionally, the service is collaborating with the National Health Insurance Service to integrate forest therapy as a means of promoting healthy lifestyles [15]. Moreover, to address social issues such as isolation and seclusion, a pilot project for forest therapy prescriptions linking forest therapy and social prescriptions is being initiated [29]. Although Korea has a forest healing infrastructure, there is no systematized model in the form of a prescribed model. Accordingly, it is necessary to develop a model for providing a comprehensive and systematic forest therapy service by linking the existing forest therapy infrastructure and medical services [28].

Therefore, the purpose of this study was to analyze the components of the forest therapy prescription model in Korea and to derive an application plan accordingly. Accordingly, the detailed goals of this study are as follows: First, the necessary components for designing a forest therapy prescription model are derived through analyses of the Korean and foreign literature and case studies. Second, through Focus Group Interviews (FGIs), expert opinions were collected to derive a specific application plan for the prescription model. This will be used as basic data to develop a systematic model for forest therapy prescriptions.

## 2. Materials and Methods

### 2.1. Research Design

**Figure 1:**
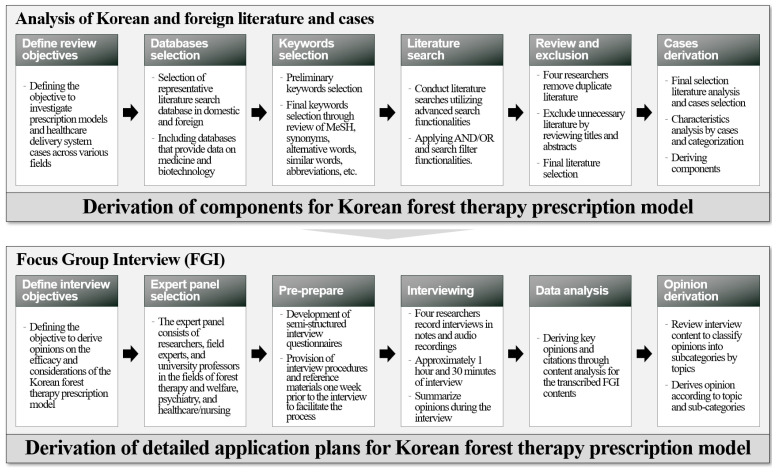
Research process diagram.

This study was conducted as an exploratory study aimed at deriving components and application plans for the design of a forest therapy prescription model. Considering the lack of specific examples of nature-based prescription models in Korea, including those for forest therapy, the Korean and foreign literature and case studies were used to analyze cases involving prescription models and healthcare service provision systems in different fields, and the basic components required to devise a forest therapy prescription model were derived. In addition, to derive a specific application plan for the forest therapy prescription model, an FGI was conducted with experts to collect and analyze opinions on the stakeholders and detailed procedures (Figure 1). To ensure the ethical validity of the research, the study was conducted with the approval of the Institutional Review Board (IRB) of Chungbuk National University (IRB Approval Number: CBNU-2024-A-0015).

### 2.2. Research Procedure

#### 2.2.1. Domestic and Foreign Literature and Case Studies

- Literature database and keyword selection

A comprehensive review of the domestic and foreign literature and case studies was conducted to derive an application plan for designing a forest therapy prescription model. First, a review was conducted of the related literature, and cases including prescription models and healthcare service provision systems for each field, such as forest therapy, medical and healthcare, exercise prescription, and smart healthcare, were derived. Data from the relevant literature and gray literature were collected using domestic and foreign literature search databases (RISS, Science ON, DBpia, National Assembly Electronic Library, MEDLINE, PubMed, EMBASE, SCOPUS, Web of Science, CINAHL, and PRISM), related ministry homepages, and reference lists. For the literature search, preliminary keywords such as “prescription”, “model”, and “development” were established, and additional keywords were derived through a review of the titles, abstracts, and original contents of the initially retrieved literature. Subsequently, the final keywords were confirmed through a meeting of four researchers, considering controlled vocabulary (MeSH), synonyms and substitutes, similar terms, and abbreviations (Table 1). Advanced search functions provided by the databases were used to enhance the precision of the literature search, including AND/OR operators and search filter functions for keywords.

- Literature search and final literature selection

**Figure 2:**
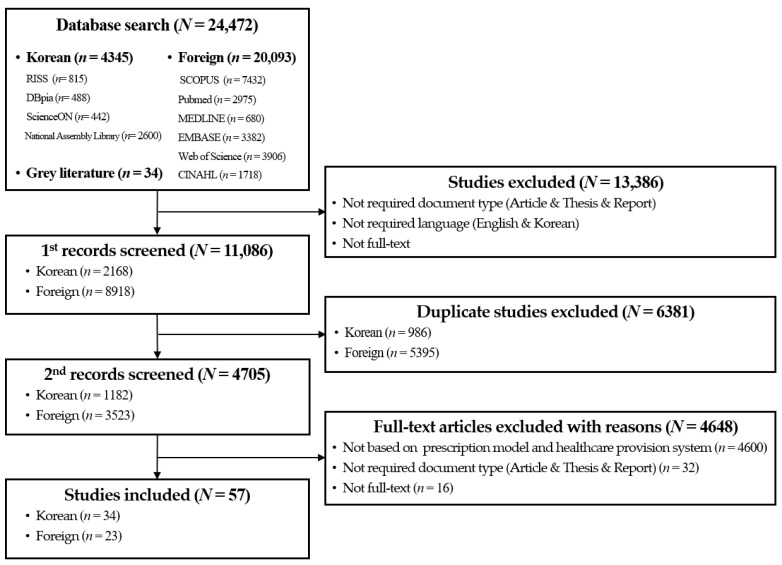
Literature selection process flowchart.

To select the relevant literature, the criteria for the literature selection were established through a meeting of four researchers. The literature selection process aimed to investigate the prescription models and healthcare service provision systems in the fields of forest therapy, medical and health services, exercise prescriptions, and smart healthcare, to analyze the characteristics of each case, and to derive application plans for forest therapy prescription models. The criteria for the literature selection were as follows: (1) articles and theses; (2) written in Korean or English; (3) studies that developed prescription models and healthcare service provision systems in the fields of forest therapy, medical and health services, exercise prescriptions, and smart healthcare; (4) studies that presented guidelines or frameworks for prescription procedures and healthcare service provision; and (5) studies that analyzed the current status of prescription models and healthcare service provision. During the literature selection process, four researchers searched domestic and foreign databases. Initially, duplicates were excluded, and irrelevant publications that did not meet the selection criteria were eliminated through a review of titles and abstracts. Subsequently, the contents of the gray literature, such as references in research reports, were reviewed, and any related publications were included in the final selection. Through this process, 57 papers were ultimately selected (Figure 2).

- Cases selection and analysis

Cases were selected that could be used to derive the components of the forest therapy prescription model by analyzing the final selected literature. The criteria for these cases were set by the researchers as follows: (1) specific cases related to the provision of prescription models and healthcare services; (2) cases of the functions, characteristics, application methods, etc., of efficient forest therapy prescriptions; and (3) cases that have similar characteristics to the forest therapy field and can be applied to forest therapy prescription. In the case selection process, four researchers reviewed the full text of the selected literature in-depth and the studies were selected through mutual review between researchers. Subsequently, five cases were selected. In the case analysis, the characteristics of the selected cases were analyzed by four researchers to derive an application plan for designing a forest therapy prescription model. Subsequently, major keywords were set for each characteristic, and items with similar characteristics were categorized. Finally, the contents of the categorized items were synthesized to derive the necessary components of a forest therapy prescription model.

#### 2.2.2. Focus Group Interview (FGI)

- FGI Expert panel selection

In this study, we attempted to derive the basic components of a forest therapy prescription model through analyses of the literature and case studies; however, it was necessary to refine these in detail and consider the expert’s perspective on the actual field, as well as the practical applicability of each aspect. Accordingly, in this study, an FGI was conducted to collect experts’ opinions on detailed procedures, stakeholders, and considerations regarding specific application plans for the design of a forest therapy prescription model. FGI is a method of collecting data on a specific topic where the data are derived through continuous discussions based on the various experiences and perspectives of a group of expert panelists [30,31,32]. Consequently, in this study, an FGI was conducted to collect detailed procedures for a forest therapy prescription model and stakeholder opinions. An expert panel was selected, including researchers, field experts, and university professors in the fields of forest therapy and welfare, psychiatry, and healthcare/nursing. The background, expertise, and experience of the experts were considered to ensure that their opinions were based on an in-depth understanding of the research topic. The criteria for the selection of the expert panel were as follows: (1) individuals with more than five years of experience in forest therapy and welfare, psychiatry, healthcare/nursing, or who participated in research and projects in related fields within the last five years; (2) individuals who understood the purpose of this study and agreed to participate in the research; and (3) individuals who could communicate without impairment of cognitive activities and judgment and who could comprehend the contents of the interview. In accordance with a study by Kim et al. [30], which suggested that it is between six and ten expert panelists provides an appropriate number, this study comprised eight expert panelists; the general characteristics of the final selected expert panel are presented in Table 2.

- Pre-prepare and interviews

A semi-structured interview protocol was developed to systematically collect opinions through the FGI. The primary components of the interview protocol were “Scope and Role of Stakeholders”, “Detailed Procedures and Effectiveness”, and “Additional Considerations” regarding the forest therapy prescription model. To facilitate the discussion, expert panelists who consented to participate in the study were provided with an e-mail containing information about the study’s topic and purpose, the interview location, the schedule, the interview protocol, and reference materials one week prior to the interview. The interview duration was approximately 1 h and 30 min, during which the opinions of the expert panelists were summarized and subsequently verified for accuracy.

- FGI data analysis

Following the FGI, a data analysis was conducted using a content analysis method for note- and audio-recordings. The content analysis method infers opinions by objectively and systematically reviewing the messages derived from the collected data [33,34,35]. In this study, during the process of deriving subcategories and determining the content for each subject from the data obtained through the FGI, it was judged that the content analysis method would make an in-depth analysis easier. The content analysis method used in this study is as follows. First, the transcription was completed by comparing and reviewing the FGI content recorded by the four researchers. The transcription was compared with the audio recordings to confirm that there was no difference in content. During this process, the Hancom Office 2020 program was used to input data on FGI content. Second, the transcription content was reviewed one sentence at a time and classified according to the topics noted in the interview paper provided in advance. Third, while reviewing the sentences, which were organized into each topics, keywords referring to core opinions were derived and classified into subcategories according to topic. Fourth, citations were selected and described in terms of the subcategories for each topic.

## 3. Results

### 3.1. Analysis of Domestic and Foreign Cases

#### 3.1.1. Analysis of the Prescription Model and Healthcare Service Provision

- Rehabilitation exercise and sports service provision model for the disabled

Kim et al. [36] proposed a health promotion model including rehabilitation exercise and sports services to promote regular exercise participation among the disabled Korean community. The model consisted of nine stages, from disability diagnosis due to injury or disease outbreak to participants’ receiving and terminating the service. to the aim was to provide this service systematically. The characteristics derived in this case were “stakeholders in service delivery”, “accessibility of the service application and delivery”, “continuous management, including of the determination and extension of prescriptions”, and a “systematic procedure for service provision”; these characteristics and the application plans are shown in Table 3.

- Forest-based customized healthcare platform

Choi and Kim [37] proposed a forest-based healthcare platform in Korea that provides customized forest therapy activities based on personal wellness records (PWR) utilizing information and communication technology (ICT). The characteristics focused on the “Database linkage of information”, “evidence-based”, “delivering a variety of content types”, and “utilizing wearable devices”, and the application plans were as shown in Table 4.

- A guidebook for a forest therapy program for depressed patients using urban forests

In a study by Shin et al. [38], face-to-face and non-face-to-face forest therapy programs using mobile applications were provided to relieve depression symptoms and improve mental health in Korean patients with depression. After the diagnosis of depression, the procedure from the prescription by a psychiatry doctor to the patient’s participation in the program and an analysis of the effects was included, and the following characteristics were derived: “utilization of online psychological testing scales”, “provision of non-face-to-face forest therapy programs”, and “management of participants through informational messaging”. The application plans based on these characteristics are as shown in Table 5.

- Non-face-to-face telemedicine and prescription cases for mental health diseases

Kim and Ko [39] analyzed telemedicine cases for individuals with developmental disabilities in domestic and international contexts and proposed a non-face-to-face treatment model and application functions. For the cases included in the literature, the necessary characteristics derived for a Doctor on Demand [40] were “non-face-to-face telemedicine and prescription”, “mental health disorder treatment spectrum”, “utilization of multiple media”, and “expert collaboration and care delivery”, as shown in Table 6.

- Oriental medicine ontology-based prescription support system

Kim et al. [41] developed a prescription support system called “Alpha” based on oriental ontology medicine to facilitate prescription decisions made according to patients’ symptoms in clinical practices in Korea. This system can be used in the process from patient symptom input to prescription recommendation, and allows for addition/subtraction and the management of results by structuring oriental medical knowledge in an ontological form. Its characteristics include the provision of “database of oriental medical symptoms and drug information”, “prescription support from database-based doctors”, the “implementation of features for coded prescriptions”, “symptom recommendation functionality”, and “prescription composition and additions”, and its application plan is shown in Table 7.

#### 3.1.2. Derivation of Components for Forest Therapy Prescription Model

The characteristics derived from the case analysis were categorized and classified into components and subcategories (Table 8). Five components were derived as being needed in the design of a forest therapy prescription model: “clear role-sharing and collaboration in system construction”, “continuous and systematic system construction”, “customized service provision system construction”, “use of various technologies and contents”, and “database-based prescription system construction”.

### 3.2. FGI Results

The opinions derived through the FGI were classified into the “scope and role of stakeholders”, “detailed procedure and its effectiveness”, and “additional considerations”. The results were summarized into subcategories and contents were arranged according to topic; key opinions and citations were presented accordingly (Table 9).

#### 3.2.1. Opinions on the Scope and Role of Stakeholders in the Forest Therapy Prescription Model

- Necessity for a middle management position

The implementation of a middle managerial role for coordinating and managing interactions between doctors, patients, and forest therapy service providers has been proposed as a necessary component for the effective execution of forest therapy prescriptions. It has been posited that middle managers who are capable of coordinating and managing prescriptions while maintaining the trust of doctors are crucial. Middle managers should possess the requisite expertise to facilitate effective communication with their doctors. It has been suggested that the establishment of trust between doctors and forest therapy facilitators through this middle management role may lead to an increase in patient referrals.

*“I think it’s a question of whether we’re going to create a model with only the infrastructure we have or think about a new infrastructure. I’m also thinking about a new infrastructure, but if you listen to everything you’re saying now, who will communicate with doctors? But doctors would never send my patient from the field to trust him and do nothing. Obviously, I think that the new thing that was not in the middle, even if it’s not a forest base, there should be someone who can trust doctors and link them.”*

(Expert A)

*“In the end, as a field expert, so when it becomes perfect, I think that the forest healing instructor will put us in the field, and instead, the areas where we communicate with doctors will have a reliable middle manager, and then the demand for patients to come to the field itself will increase.”*

(Expert A)

Furthermore, the middle manager in the forest therapy prescription model was proposed to have several roles: facilitating patient transportation to the site following the prescription, as well as helping the patient during the program’s execution and assisting in the management process; liaising between doctors and forest therapy service providers to address unforeseen circumstances or participant difficulties during the program; and optimizing the efficacy of forest therapy prescriptions by providing feedback to doctors regarding program modifications or changes in patient status.

*“The middle manager is the person who assists in forest therapy prescriptions. Their role would be to help them make forest therapy service reservations, help them move to the forest therapy place, move together on the same day, change during the program, or arrange contacts in the middle and reset them again if the participants were having a hard time.”*

(Expert D)

*“There may be medical institutions that need middle managers, and there are only a few mental health doctors who are familiar with forest therapy, so it is likely that there should be middle managers before the doctor identifies the structure of the forest therapy prescription.”*

(Expert E)

It has been proposed that the use of middle managers in the forest therapy prescription model could enhance the efficiency of the prescription, implementation, and administration processes. However, it is imperative to consider the potential increase in costs associated with human resource allocation.

*“It would definitely be convenient and good for the medical field to have such a person, but for forest therapy, it would be more expensive to receive forest therapy services as the middle manager is included, so it is important how to prove that the cost is lower than the existing treatment cost.”*

(Expert E)

- Securing the expertise of forest healing instructors

It has been posited that securing the expertise of forest healing instructors is crucial for the implementation of forest therapy prescriptions. Furthermore, forest healing instructors must possess fundamental qualifications to effectively ensure the doctors’ prescriptions are fulfilled. It was proposed that these qualifications should be predicated on relevant academic disciplines, such as nursing, health sciences, and psychology, or alternatively on the possession of counseling credentials or a record of completing supplementary education.

*“Basically, whether you have a base for nursing, are you qualified for counseling, or have studied additional counseling, I think it would be better to think about this because only a forest healing instructor with such a base can carry out forest therapy prescriptions.”*

(Expert G)

*“Considering that they are internal employees of forest therapy service providers, there are nursing majors, health majors, and psychology majors among them, so it would be good to consider these areas and put them in.”*

(Expert B)

Furthermore, it was concluded that forest healing instructors should enhance their expertise by specializing in specific subject groups, diseases, and therapeutic methods and activities. Given that current forest healing instructors come from diverse backgrounds (including nurses, psychotherapists, teachers, and forest therapy majors), this opinion suggests that such specialization could enable the development of tailored forest therapy programs and clearly define the target treatments.

*“Currently, there are various bases among forest healing instructors, including nurses and teachers. There are psychotherapists, some people have majored in forest therapy, and some people are familiar with forest therapy factors and environments, so I think it’s a good idea to distinguish the current forest healing instructors by job group. Then, forest therapy programs or diseases that can respond to will be embodied.”*

(Expert C)

Analogous to the evaluation of medical quality in the United States, a quality control plan has been proposed to ensure continuous competency evaluations are carried out, and to ensure the management of forest healing instructors involved in forest therapy prescriptions. This plan stipulates that if certain criteria are not met through regular evaluation, re-education or re-certification would be required.

*“There’s a quality control. The U.S. and other countries evaluate him once a year to see if he is really doing right, and if he deviates from the percentage, he will fail and have to re-qualify.”*

(Expert H)

#### 3.2.2. Opinions on the Detailed Procedures and Effectiveness of the Forest Therapy Prescription Model

- Consideration of additional procedures

It has been concluded that additional detailed procedures warrant consideration for the development of efficacious forest therapy prescriptions. Therefore, it is imperative to objectively validate the effectiveness of forest therapy prescriptions by comparing pre- and post-program outcomes using standardized psychological scale tests. Furthermore, a preliminary consultation phase was proposed to identify the subjects’ needs and preferences to develop personalized forest therapy prescriptions, as well as a procedure for continuous management and monitoring following forest therapy prescriptions. In terms of protecting patient privacy, the handling of personal information related to patient data and prescription-sharing were identified as critical issues.

*“It should be checked once through the psychological scale at the time of reception and visit, so even after the program is carried out, the procedure for comparing the psychological scale pre-post should be considered.”*

(Expert F)

*“When we do continuous management (monitoring), we need to think about who will take charge of it, and I hope that the concept of continuous management will be transferred to the center after the program, how the forest healing instructor will manage it, and in this way, the concept of continuous management (monitoring) will be concrete.”*

(Expert F)

*“People who provide forest therapy programs should have a basic understanding of people with mental health disorders. So I think there should be a preliminary step in understanding the subject before the program.”*

(Expert G)

*“Sharing personal information will not be easy. Here is sharing of patient information and prescriptions. We need to think about how to solve this problem. In this regard, I hope that parts of the personal information consent form will be made well, and the contents of the personal information consent process will be included in advance.”*

(Expert F)

- Method for providing forest therapy services

It has been proposed that the methods of individual and group programs should be considered to provide effective forest therapy services. While one-on-one customized programs are effective, in that they can be tailored to suit individual characteristics and needs, the psychological stability and synergistic effects achieved through social interactions and collaborative activities in group programs have also been identified as significant factors. Notably, in the case of individuals with mental health disorders, it has been emphasized that group programs have a positive effect on mitigating the perception of being patients and reducing the psychological burden through community-based activities.

*“Personally, if you prescribe a patient, a prescription or forest therapy program should be operated as a 1:1 response structure, but in the actual field, it is operated as a family unit for organizations, so I think it is necessary to introduce it in a way when applying it in the field. Otherwise, the field has no choice but to collect people with the same type of disease and run the program. In a way, it can be seen as a PT (Personal Training) concept.”*

(Expert B)

*“If you are able to live your daily life to the extent that you take medicine for mental health disorders, even if it is not necessarily a 1:1 form, the program has another effect, even when it is a group program. There may be negative aspects that come from being treated specially or being perceived as too ill. Being buried a little together is also an advantage, and there are some areas where synergy can be exerted while working together, so I think it would be better to consider a group that is not necessarily 1:1.”*

(Expert C)

It was posited that a plan was necessary to address accessibility issues in forest therapy services. The limited accessibility of national forest therapy facilities was identified as a concern, and to mitigate this, it was proposed to actively utilize healing forests or green welfare centers in proximity to urban centers. Through this approach, it was suggested that an environment could be created wherein forest therapy programs can be more readily integrated into daily life, thereby increasing participation.

*“To tell you one thing about accessibility, accessibility is the biggest problem with national forest therapy facilities. So, Seoul has about seven green welfare centers, so I think it would be better to operate them in the form that targets them. In the end, I think we should approach it as a daily type, and there are accommodation types, but in the end, I think we should actively use facilities, such as nearby healing forests.”*

(Expert B)

- Stepwise application of models

It was proposed that the forest therapy prescription model should not be confined to the medical field when relating to mental health disorders, but should be progressively expanded through a systematic approach encompassing stress management, health promotion, and the prevention and management of mental health disorders. In the initial stages, health-promotion-oriented programs that are accessible to the general population could be implemented to support overall health maintenance and stress management. In subsequent stages, it has been suggested that medical institutions, particularly those specializing in mental health, could develop customized in-depth programs for specific individuals requiring management through prescription. Furthermore, recommendations were made to establish a more integrated and sustainable model through collaboration between medical institutions and community-based mental health welfare centers. It was emphasized that forest therapy programs can be utilized not only by patients with mental illnesses, but also by those with chronic conditions through cooperation with local communities. Additionally, it was proposed that these programs should be expanded into a nationwide health management initiative in conjunction with the National Health Insurance Corporation’s support system or the National Health Promotion Comprehensive Plan.

*“I think it is necessary to apply it step by step to the forest therapy prescription model. For example, I think there are about three stages: the initial stage, the advanced stage, and the stable stage. In the early stages, it will be a stage in the health promotion area that anyone can do, and in the other stages, I think it will be necessary to create a step-by-step model so that someone special can do it.”*

(Expert C)

*“I think it can be divided into choosing the concept of medicine or treatment and aiming for health promotion by a little tone down. When it comes to the medical field, I think that first being diagnosed with depression in the department of psychiatry and prescribing is clearly carried out at the medical center, and it can be a health welfare center to communicate about health promotion.”*

(Expert A)

*“Mental health welfare centers are different from the hospital system because they enter community health. Hospitals have prescriptions in the system, but mental health welfare centers are a way to intermediate connections to certain institutions or facilities in the form of “center prescriptions” in the community.”*

(Expert F)

*“It is the use of a health welfare center as a concept of how to live together with the community, and doctors are often a public-oriented institution that does not only meet and prescribe, but also programs, counsels, visits, and plays such a role. But not only here, but also if you think of people with mental health diseases, I think it is right to cooperate with the Mental Health Welfare Center to build a model.”*

(Expert G)

*“In the area of mental health, if you want to target people with diseases and do something like the health living support system, you can also link it with subsidies to the National Health Insurance Service by targeting people with chronic diseases (such as high blood pressure and diabetes). A broad concept for the entire nation may also require efforts to include it in the Comprehensive National Health Promotion Plan mentioned earlier.”*

(Expert G)

#### 3.2.3. Opinions on the Additional Considerations of Forest Therapy Prescription Models and Insurance Linkage Plans

- Implementation of technology with regard to scalability

It was proposed that the implementation of forest therapy prescriptions within institutional frameworks and their integration with insurance systems necessitate the establishment of a management system utilizing a digital platform. Furthermore, the utilization of forest therapy programs incorporating AR and VR technologies, as well as the assessment of forest therapy efficacy using biomarkers or wearable devices, were identified as crucial technical components. The importance of considering the use of these digital technologies for potential integration with national systems was emphasized.

*“There is a place called the Korea Social Security information Service, which is operated by doctors operated by the National Health Insurance Service and the Health Insurance Review and Assessment Service in the field of systems and welfare, and I would like to say that in the end, the platform should not be distributed by the Korea Forest Service, but should be considered that it can be transferred into the national system in the long run.”*

(Expert G)

*“Additionally, what I expect a little is that I hope you put in the part that uses digital platforms or wearable devices. I think they are the direction of health care in terms of technology, and I hope the overall frame goes like that.”*

(Expert A)

- Necessity for specialized scales in the field of forest therapy

It was proposed that a specialized scale is necessary to measure the specific effects of forest therapy. In addition to existing mental health assessment instruments, it was suggested that a specialized psychological scale or evaluation protocol reflecting the characteristics and effects of forest therapy should be developed to evaluate the efficacy of forest therapy as distinct from other therapeutic interventions.

*“Since there are already too many forest therapy programs these days, such as depression and anxiety, I hope that the unique scale of the forest therapy field, which reflects forest therapy characteristics, will be joined with the psychological scale, rather than just a general scale from an ideological point of view, and more meaningful results will be obtained. Rather, I thought it would be nice to find and put in not only psychological tests but also things that can be combined with something a little different.”*

(Expert F)

- Forest therapy prescription components

Therefore, it is imperative to consider the patient’s physical functioning. Given that the forest therapy program is conducted in an external environment, specifically in a forest, the range of potential physical activities the patient can carry out must be evaluated. For instance, it is essential for a doctor to assess whether the patient experiences mobility limitations or requires the use of a wheelchair and subsequently prescribe a program that aligns with the patient’s physical capabilities. This assessment would serve as the foundation for ensuring patient safety and facilitating effective program implementation.

*“The doctor should figure out the patient’s body function. And you can check how much activity is possible in this patient’s area of activity. For example, a doctor should designate the degree of activity in the areas where this person has to ride a wheelchair, there is no problem walking, and he can hike a mountain.”*

(Expert D)

Specific elements of the prescribed forest therapy program should be delineated. These elements should be articulated in detail, including the activities involved, the location of the therapy, duration of the therapy, session structure, and the frequency of the forest therapy program. Doctors should specify the content and format of the program based on a comprehensive understanding of forest therapy, ensuring that the program can be conducted under doctor’s supervision.

*“If it becomes a forest therapy prescription, the forest environment will be important, and the doctor will have to specify what programs will be operated in it. If the doctor says he doesn’t know about the program, insurance won’t be applied. Basically, most treatments should be very basic for doctors to know about them. And it should be an area that doctors can do. It doesn’t matter if forest therapy is done for health care.”*

(Expert D)

*“In order to be medical area, you will have to designate a place. Because it doesn’t make sense if the doctor says he didn’t know when a problem broke out in here. So the doctor has to designate everything from this program to the right one so that the doctor can take responsibility for it. Also, since there is a premise that a doctor is responsible, a doctor is responsible for the prescription, but from a doctor’s point of view, if the prescription I gave can be modified and changed by someone, it does not fall under the medical field.”*

(Expert D)

*“The forest therapy prescription should come out, for example, This person should receive a forest therapy program for depression several times a week for several months because depression is mild. It’s right to appear in the form of “what to do.” “There should be an order at the end of what to do.”*

(Expert E)

## 4. Discussion

This study was conducted as an exploratory study to determine the necessary components and application plans for forest therapy prescription models. To this end, cases including Korean and foreign prescription models and healthcare service delivery systems were analyzed, and an FGI was conducted including experts in the fields of forest therapy and welfare, psychiatry, and healthcare/nursing. Through this, five necessary components of the design of a forest therapy prescription model were derived, and specific application plans focusing on six topics were derived for effective forest therapy prescriptions.

### 4.1. Application Plans

Accordingly, the application plans for a forest therapy prescription model are as follows.

First, middle managers are essential to efficient forest therapy prescriptions. Middle managers for Korean and foreign prescriptions include hospital and medical institution coordinators [42,43], care coordinators [44,45], and link workers [46], who oversee prescription support, on-site connection, and patient management. To participate in the forest healing program in Korea, individuals are required to make reservations and apply through an online reservation system [47], selecting from pre-existing programs. These constraints necessitate that users verify their information directly to identify a suitable program, which can lead to challenges in obtaining a program tailored to an individual’s health condition or specific objectives. Furthermore, the inherent variability of forest environments means that key forest healing resources and environmental characteristics that could enhance the healing effect may differ across locations [48]. This variability presents practical challenges for doctors in identifying and prescribing treatments involving these characteristics. In this context, forest therapy prescriptions aim to offer a personalized program for health promotion and symptom improvement by fostering collaboration between the expertise of prescribers (doctors), service-providers (forest healing instructors), and middle managers. Consequently, middle managers in forest therapy prescriptions should facilitate communication between doctors, patients, and forest therapy service-providers, support the reservation and execution of customized programs based on doctor’s prescriptions, and address potential issues that may arise during the program. Furthermore, it is essential to develop expertise and technology that can be associated with forest healing resources and environmental characteristics specific to each location. This approach aims to improve the therapeutic effects of forests by collaborating with forest healing service-providers.

Second, an integrated management system utilizing a digital platform is essential for the efficient implementation of forest therapy prescriptions and continuous accumulation of data. It is imperative to establish a system that will contain prescription history and allow for effective data management by integrating patient health and prescription outcome data into a unified platform. Specifically, it is crucial to monitor changes in health status using wearable devices and biomarkers that can assess the severity of symptoms [37,49], and to establish a system capable of objectively evaluating the efficacy of a forest therapy program. Furthermore, there is a need for a system in which patients, doctors, middle managers, and forest therapy service-providers can interact to discuss prescription support and patient management. Such a system can facilitate seamless information-sharing and collaboration among patients, doctors, middle managers, and forest therapy service-providers.

Third, to optimize the therapeutic benefits of forest environments, it is imperative to develop and quantify a standardized database of forest healing resources. The efficacy of forest therapy is contingent upon both physical attributes, such as the forest’s structure, ecological characteristics, area, and locational factors, as well as environmental elements, including volatile organic compounds (NVOC) in the air, fragrance, thermal comfort, and visual comfort [50]. Human perception of the forest environment is mediated through five senses—sight, smell, hearing, touch, and taste—which collectively contribute to the healing experience [51]. Notably, activities such as meditation, walking, and breathing within the forest may enhance therapeutic outcomes when integrated with cognitive behavioral therapy [52]. Engaging in diverse forest healing resources can facilitate psychological relaxation and stability, potentially leading to health benefits through enhanced immunity [53]. Given the variability of forest environmental elements across different locations, it is essential to establish and quantify a standardized database [54,55,56] for forest healing resources. The application of this database to forest therapy prescription models, in conjunction with digital platforms, will aid doctors in prescribing personalized forest therapy programs and effectively disseminate information to forest healing instructors and middle managers.

Fourth, for the systematic implementation of forest therapy prescriptions, detailed procedures for each step must be clearly delineated. To this end, the forest therapy prescription stage should be designed as a procedure that encompasses treatment and diagnosis, forest therapy prescriptions, the reservation and execution of forest therapy programs, an evaluation of their effectiveness, the termination of prescriptions, and follow-up management. In this process, a program should be prescribed that considers the patient’s range of physical activity according to the doctor’s diagnosis, thereby providing customized forest therapy services that reflect health conditions. Furthermore, measures should be established to safeguard patients’ personal and sensitive information. To analyze the effects of forest therapy prescriptions, it is necessary to establish the ability to continuously accumulate data on clinical effects by utilizing specialized psychological scales applicable in the field of forest therapy [57]. Upon completion of the forest therapy prescription, a system should be established to provide information that enables patients to continue their own forest therapy activities and receive additional prescriptions if necessary. This sustainable management system will contribute to maintaining the long-term effects of forest therapy prescriptions and improving patients’ quality of life.

Fifth, the forest therapy prescription model should be approached in stages, such as health promotion and medical areas, so that it can be customized according to the subject’s situation. It can be divided into health promotion, aimed at disease prevention and management through promoting stress reduction, psychological stability, and physical activity to relatively healthy people, and medical areas with therapeutic purposes, focusing on the management of physical and mental diseases or chronic diseases. This step-by-step approach can establish a continuous management system encompassing health promotion, disease prevention, management, and treatment, and maximize the therapeutic effect through customized prescriptions. Furthermore, the use of differentiated models in each sector will increase the feasibility of integrating patients’ insurance and providing institutional support for forest therapy and ultimately disseminate the value of forest therapy within the public health and medical system.

Sixth, it is imperative to establish a system that clearly delineates the roles of various stakeholders, including patients, prescribers (doctors), practitioners (forest healing instructors), and middle managers, for forest therapy prescriptions and allocate responsibilities accordingly. In particular, a link with medical services is required, and prescriptions must be made by trained medical professionals [28]. Doctors should assume responsibility for prescribing customized forest therapy programs based on patients’ health status and determine the necessity of additional prescriptions. Furthermore, forest healing instructors who are affiliated with forest therapy service-providers should possess the requisite expertise and qualifications to implement the prescribed program effectively in the field. To achieve this, it is necessary to enhance expertise through the specialization of forest healing instructors in various subjects and diseases, continuing their education for increase their capacity [58], and implementing a continuous competency evaluation and management plan akin to medical quality evaluations [59].

### 4.2. Study Significance

This study is meaningful in that it provides basic data for the future development of a Korean forest therapy prescription model by analyzing existing prescription models and healthcare service provision systems and deriving the components and application plans of effective forest therapy prescriptions through an FGI including experts in the field. In particular, to supplement the results of the Korean and foreign literature and case analyses, specific measures regarding the application of Korean forest therapy prescriptions were discussed, collecting opinions based on the expertise and experience of researchers, university professors, and field experts in each field. Through this, the foundation for customized forest therapy prescriptions was laid. These results may contribute not only to the application and spread of the Korean forest therapy prescription model, but also to the establishment of strategies to apply nature-based prescription practices in other countries.

### 4.3. Study Limitations and Suggestions

This study has the following limitations. First, it may have limited applicability to other countries because its analyses were based on the prescription model and healthcare service delivery system of Korea. Second, as an exploratory study, this study was limited in that it did not consider the perspective of patients or the general public using forest therapy prescriptions because it focused mainly on the literature research and expert opinions. Third, the expert panel was restricted to the domains of forest therapy and welfare, psychiatry, and healthcare/nursing, and therefore did not encompass a wide range of perspectives.

Suggestions for follow-up studies regarding the application and spread of the Korean forest therapy prescription model are as follows. First, it is imperative to define the role of middle managers within the context of the forest therapy prescription and to undertake subsequent studies aimed at conducting a detailed job analysis. Second, a follow-up study on the design and empirical application of the forest therapy prescription model through a field application involving patients and users is required. Third, Third, to establish strategies for managing chronic and physical diseases, as well as for health promotion—including quality-of-life improvement, stress management, and improvements in mental health disorders—follow-up studies are needed that will gather in-depth opinions from multidisciplinary groups.

## 5. Conclusions

This study was an exploratory study to derive the necessary components and application plans for a systematic model to introduce forest therapy prescriptions in Korea through an analysis of the domestic and foreign literature, case studies, and an FGI with experts. The findings of the study identified several critical factors for designing and empirically applying the forest therapy prescription model, including the delineation of the scope and roles of stakeholders, the need for detailed procedures at each stage, a phased approach to health promotion and the medical arena, and the utilization of digital platforms. These results will contribute to establishing a prescription system for forest therapy and medical services and can be used as basic data for introducing a system of forest therapy prescriptions.

## Figures and Tables

**Table 1:** Final keywords selected for the domestic and foreign literature search.

Categories	Keywords
Korean	“처방 (prescription)” OR “건강 (health)” AND “모델 (model)” OR “시스템 (system)” OR “모형 (structure)” OR “서비스 (service)” OR “가이드라인 (guideline)” OR “지침 (guideline)” OR “플랫폼 (platform)” AND “구상 (design)” OR “구축 (build)” OR “개발 (development)” OR “설계 (design)”
Foreign	“prescription” OR “prescribing” OR “Rx” OR “health” AND “model” OR “system” OR “structure” OR “framework” OR “mechanism” OR “platform” AND “build” OR “building” OR “development” OR “design” OR “implement” OR “main elements” OR “plan” OR “guideline”

**Table 2:** General characteristics of the FGI expert panel.

No	Fields	Panels	Gender	Affiliation (Location)	Years of Experience
1	Forest therapy and welfare	Expert A	Female	Research institution belonging to the Korea Service (Seoul)	25
2		Expert B	Female	Public institution belonging to the Korea Service (Daejeon)	8
3		Expert C	Female	National healing forest belonging to the Korea Service (Kangwon)	11
4	Psychiatry	Expert D	Male	Mental health clinic (Seoul)	24
5		Expert E	Male	Mental health clinic (Seoul)	22
6	Healthcare/nursing	Expert F	Female	Public institution belonging to the Ministry of Health and Welfare (Chungbuk)	30
7		Expert G	Female	Dept. of nursing, University (Incheon)	17
8		Expert H	Male	Dept. of Basic science, University (Chungbuk)	14

**Table 3:** Characteristics of a rehabilitation exercise and sports service provision model for the disabled and the application of the forest therapy prescription model.

Categories	Characteristics	Application Plan
Stakeholders in service delivery	Clear delineation of roles among patients, prescribers (doctors), and rehabilitation exercise and support center instructors (service providers), considering the system from both consumer and provider perspectives.	It is imperative to clearly delineate the roles of stakeholders, including patients, prescribers (doctors), and forest healing instructors, and to establish a system for sharing responsibilities.
Securing accessibility for service application and delivery	Enhancing the accessibility of the services is possible through the utilization of community centers in the vicinity, allowing for service applications; non-face-to-face service applications are also available for those who face difficulties visiting in person. Furthermore, accessibility is improved through service provision via rehabilitation exercise and support centers.	To enhance access to forest therapy, it is necessary to develop a connection between forest therapy locations and users through collaboration with forest therapy service providers and affiliated institutions.
Continuous management, including prescription determination and extension	Reassess health status upon the completion of prescription, reviewing additional diagnoses through various tests, the feasibility of rehabilitation exercise prescriptions, and exercise limitations, to determine the necessity for service extension and the provision of ongoing information and support.	It is essential to ascertain the availability of appropriate forest therapy prescriptions through monitoring, and to provide education and information that will enable patients to continue their forest therapy activities independently after the prescription period, as well as to establish a system for obtaining additional prescriptions when necessary.
Systematic procedure for service provision	Implement systematic services through clearly defined nine-step procedures spanning from their initiation to their conclusion.	A well-defined, step-by-step procedure encompassing the initial diagnosis, prescription, implementation, evaluation, termination, and follow-up management should be designed to ensure a systematic, user-centered forest therapy prescription process.

**Table 4:** Characteristics of forest-based customized healthcare platform and application of forest therapy prescription model.

Categories	Characteristics	Application Plan
Database linkage of information	Basic health information, health status measurement results, and customized forest therapy activities can be efficiently viewed and managed through integrating the database on a single platform.	In the forest therapy prescription model, patient health information and prescription result data should be integrated into a unified platform to establish a system that facilitates easy access and utilization for users and providers.
Evidence-based	Provision of personalized forest therapy activities based on evidence-based health status measurement parameters.	The system should be configured to provide a customized forest therapy prescription program in accordance with a doctor’s diagnosis based on scientific evidence.
Delivering a variety of content types	Five categories of forest therapy content were presented based on diverse health information, including the physical, psychological, and nutritional aspects, with psychological and therapeutic content incorporated according to preference indices.	In the forest therapy prescription model, the contents of a customized forest therapy prescription program should be developed with consideration of the patient’s health status, preferences, and requirements.
Utilizing wearable devices	Monitor forest exercises and provide health information feedback based on data collected via wearable devices.	It is essential to monitor the outcomes of forest therapy prescriptions using wearable devices, provide appropriate feedback, and establish an additional prescription adjustment system.

**Table 5:** Characteristics of the forest therapy program guidebook for depressed patients using urban forests and application of the forest therapy prescription model.

Categories	Characteristics	Application Plan
Utilization of online psychological testing scales	Psychological scales pertaining to mental health disorders (depression, anxiety, stress, etc.) can be efficiently administered and evaluated via online surveys integrated with databases.	In forest therapy prescriptions, a system should be established to digitize psychological-scale items and integrate them with the platform database, enabling doctors and patients to readily access and review tests and results.
Provision of non-face-to-face forest therapy programs	Forest therapy programs can be implemented through video content that is prescribed and accessed via mobile applications.	Based on the patient’s health status, forest therapy programs should be implemented through remote forest therapy prescriptions, with monitored effects and a system for communication with doctors or program managers when necessary.
Management of participants through informational messaging	The effective management of program operations and participants should be ensured through the provision of program participation confirmation, scheduling, and precautionary information offered via telephone and text-based communication.	To enhance participation rates in forest therapy prescriptions, a management system that incorporates functions for delivering notifications and guidance to patients should be developed.

**Table 6:** Characteristics of non-face-to-face telemedicine and prescription cases for mental health diseases and application of the forest therapy prescription model.

Categories	Characteristics	Application Plan
Non-face-to-face telemedicine and prescription	Video-based treatment, consultations, and prescriptions are feasible through smartphones, tablets, personal computers, etc., prescriptions can be transmitted to pharmacies, or existing prescriptions can be renewed.	Non-face-to-face treatment and prescription functions should be implemented for patients who face difficulties in accessing forest therapy prescriptions, establishing a system in which healthcare providers and patients can interact regardless of location and time.
Mental health disorder treatment spectrum	Provide prescriptions for the treatment and prevention of various mental health disorders, including stress, anxiety, interpersonal difficulties, depression, mood alterations, substance use disorders (alcohol and tobacco), and eating disorders.	It is necessary to implement multifaceted approaches, such as prevention, counseling, psychological interventions, and education for mental health disorders, such as depression and stress.
Utilization of multiple media	Deliver psychological counseling and educational content utilize technologies such as video and virtual reality and implement long-term programs through features that promote user engagement and interest.	Augmented Reality (AR) and Virtual Reality (VR) technologies should be incorporated into non-face-to-face forest therapy programs to provide immersive content and enhance patient engagement and active participation.
Expert collaboration and care delivery	Collaborate with experts such as psychotherapists and social workers to provide patients with necessary Online Therapy and care and support patient care through guidance resources such as Navigator.	In the forest therapy prescription model, a collaborative system among forest healing instructors, psychotherapists, and social workers should be established to enable personalized treatment and the long-term care of patients.

**Table 7:** Characteristics of oriental medicine ontology-based prescription support system and its application to the forest therapy prescription model.

Categories	Characteristics	Application Plan
Database of oriental medical symptoms and drug information	Database of oriental medical symptoms and drug information to be integrated onto the platform.	It is imperative to establish a database of the types, effects, and activities of forest therapy programs and integrate this into the prescription system.
Prescription support from database-based doctors	Assist in prescription determination by recommending pathology and treatment options upon the input of patient symptoms.	When inputting a patient’s health status, it is essential to develop a system that recommends an appropriate forest therapy program and supports its composition.
Implementation of features for coded prescriptions	Storage of frequently utilized prescriptions in coded format for reuse in similar patient cases.	It is necessary to streamline the iterative prescription process by storing and managing standardized forest therapy programs in the form of coded prescriptions.
Symptom recommendation functionality	Augmentation of the prescription process through automatic recommendation of related symptoms beyond those initially entered	When selecting forest therapy activities, it is crucial to establish a function that can automatically recommend additional therapy activities or related programs in addition to existing information.
Prescription composition and additions	Provision of functionality to modify, supplement, or remove components of the selected prescription	When constructing a forest therapy program, it should be designed to allow for the addition or deletion of activity content or modification of the program according to specific conditions.

**Table 8:** Components and sub-categories when designing a forest therapy prescription model.

Components	Sub-Categories	Contents
Clear role-sharing and collaboration system construction	Establishing a role-sharing system for each stakeholder	Clarify the roles of interested parties, such as patients, prescribers (doctors), and forest healing instructors. Establish a role-sharing system for safe and effective operations.
	Customized management through multidisciplinary collaboration	Establish a cooperative system between forest healing instructors, psychotherapists, and social workers. Establish patient-specific care and long-term management.
	Connection between local forest therapy service-providers and community centers	Establish a cooperative system with forest therapy service providers and similar institutions to enhance access to forest therapy. Establish a connection between forest therapy sites and users.
Continuous and systematic system construction	Prolonging and providing ongoing management of prescriptions	Monitor patients to determine appropriate forest therapy prescriptions. Provide education and information to help patients continue voluntary forest therapy activities even after the prescription is terminated. Establish a system for patients to receive additional prescriptions if necessary.
	Systematic service provision procedure	Design clear step-by-step procedures that lead to the initial diagnosis, prescription, execution, assessment, termination, and follow-up. Guarantee a user-centered systematic forest therapy prescription service.
	Application of forest therapy digital prescription platform	Digitize inspection items and link them to the platform database. Establish a system that allows doctors and patients to easily check results.
	Implement features for efficient prescription	Store and manage standardized forest therapy programs in the form of promised prescriptions. Streamline repetitive forest therapy procedures.
Customized service provision system construction	Provision of evidence-based content	Provide a customized forest therapy prescription program based on scientific evidence.
	Scope of treatment for physical and mental illnesses	Establish a continuous monitoring system through access to exercise, nutrition, etc., for chronic diseases such as high blood pressure. Introduce multifaceted approaches such as prevention, counseling, psychology, and education for mental health diseases such as depression and stress.
	Implement symptom-specific recommendations	Establish a function that can automatically recommend additional therapy activities or related programs in addition to existing information when selecting forest therapy activities.
	User-centric content delivery	Provide customized forest therapy prescription programs considering the health status, preferences, and needs of patients.
	Non-face-to-face forest therapy programs	Provide non-face-to-face forest therapy prescription and monitoring according to the patient’s health requitements. Establish a system to communicate with doctors and managers if necessary.
Use of various technologies and contents	Leverage a variety of delivery media	Provide immersive content through non-face-to-face forest therapy programs such as AR and VR, encouraging patients to be interested and actively participate.
	Use of wearable devices	Implement and monitor forest therapy prescriptions using wearable devices, and provide appropriate feedback. Establish an additional prescription-adjustable system based on physical indicators.
Database-based prescription system construction	DB information	Establish a database including the types, effects, and activities of forest therapy programs. Continuously accumulate and manage prescription data on various subjects and symptoms. Link prescription systems and digital platforms
	Link information to digital platforms	Integrate platforms containing patient health information and data on the prescription and results. Establish a system that makes this database easy for users and providers to access and utilize.

**Table 9:** Sub-categories and contents of the FGI by application plans for the design of a forest therapy prescription model.

Topics	Sub-Categories	Contents
Scope and role of stakeholders	Necessity for a middle management position	Requirement for a professional middle manager to facilitate communication with doctors. Development of a role coordinating and managing forest therapy prescriptions and practices. Necessity to consider the cost implications of additional workforce utilization.
	Securing the expertise of forest healing instructors	Evaluation of the qualifications of forest healing instructors who implement forest therapy prescriptions. The necessity to enhance the competencies of forest healing instructors. Requirement for forest healing instructors to specialize according to their areas of expertise.
Detailed procedures and effectiveness	Consideration of additional procedures	Implementation of a comparative analysis pre- and post-forest therapy program. Necessity for pre-consultation procedures to ensure patients comprehend the subject. Requirement for ensuring continuous management (monitoring) during procedures. Mandatory process for obtaining consent regarding the use of patient’s personal information (sensitive data).
	Method for providing forest therapy services	Consideration of operational methodologies in the provision of forest therapy programs. Necessity to address accessibility concerns.
	Stepwise application of models	Categorization into healthcare and medical care domains.
Additional considerations	Implementation of technology with regard to scalability	Integration of digital platforms and wearable devices. Requirement for a platform to interface with national systems.
	Necessity for specialized scales in the field of forest therapy	Consideration of testing protocols to determine the specific effects of forest therapy.
	Forest therapy prescription components	Evaluation of the patient’s range of physical activity. Designation of specific forest therapy programs by doctors.

## Data Availability

The original contributions presented in this study are included in the article. Further inquiries can be directed to the corresponding author.

## References

[1] Kim J.S. (2024). To Find a Way in the Healing Industry.

[2] Kondo M.C., Fluehr J.M., McKeon T., Branas C.C. (2018). Urban green space and its impact on human health. Int. J. Environ. Res. Public Health.

[3] Brymer E., Freeman E., Richardson M. (2019). One health: The well-being impacts of human-nature relationships. Front. Psychol..

[4] Corazon S.S., Sidenius U., Poulsen D.V., Gramkow M.C., Stigsdotter U.K. (2019). Psycho-physiological stress recovery in outdoor nature-based interventions: A systematic review of the past eight years of research. Int. J. Environ. Res. Public Health.

[5] Braubach M., Kendrovski V., Jarosinska D., Mudu P., Andreucci M.B., Beute F., Davies Z., de Vries S., Glanville J., Keune H. (2021). Green and Blue Spaces and Mental Health: New Evidence and Perspectives for Action.

[6] Pneumokur. https://pneumokur.de.

[7] James J.J., Christiana R.W., Battista R.A. (2019). A historical and critical analysis of park prescriptions. J. Leis. Res..

[8] Park S.J., Kim G.W. (2021). Overseas Cases of Medical Linkage Services Using Forest Resources.

[9] Migl W., Mathis H., Spencer M., Hernandez R., Maddock J.E. (2024). A Scoping Review of Nature Prescriptions Offered by Healthcare Providers. J. Public Health Emerg..

[10] PaRX. https://www.parkprescriptions.ca.

[11] Golden Gate Institute https://www.parkrx.org.

[12] Kondo M.C., Oyekanmi K.O., Gibson A., South E.C., Bocarro J., Hipp J.A. (2020). Nature prescriptions for health: A review of evidence and research opportunities. Int. J. Environ. Res. Public Health.

[13] Nguyen P., Astell-Burt T., Rahimi-Ardabili H., Feng X. (2023). Effect of nature prescriptions on cardiometabolic and mental health, and physical activity: A systematic review. Lancet Planet. Health.

[14] Frumkin H., Bratman G.N., Breslow S.J., Cochran B., Kahn P.H., Lawler J.J., Levin P.S., Tandon P.S., Varanasi U., Wolf K.L. (2017). Nature contact and human health: A research agenda. Environ. Health Perspect..

[15] Korea Forest Service https://www.forest.go.kr.

[16] Stier-Jarmer M., Throner V., Kirschneck M., Immich G., Frisch D., Schuh A. (2021). The psychological and physical effects of forests on human health: A systematic review of systematic reviews and meta-analyses. Int. J. Environ. Res. Public Health.

[17] Rosa C.D., Larson L.R., Collado S., Profice C.C. (2021). Forest therapy can prevent and treat depression: Evidence from meta-analyses. Urban For. Urban Green..

[18] Kwon M.G., Yeon P.S., Min G.M. (2023). The improvement effect of stress through forest activity: A systematic review and meta-analysis. J. Korean Inst. For. Recreat..

[19] Chae Y.R., Lee S.H., Jo Y.M., Kang S.Y., Park S.Y., Kang H.Y. (2021). The effects of forest therapy on immune function. Int. J. Environ. Res. Public Health.

[20] Yeon P.S., Jeon J.Y., Jung M.S., Min G.M., Kim G.Y., Han K.M., Shin M.J., Jo S.H., Kim J.G., Shin W.S. (2021). Effect of forest therapy on depression and anxiety: A systematic review and meta-analysis. Int. J. Environ. Res. Public Health.

[21] Kim A.R., An K.W. (2021). Physiological and psychological changes of forest experience programs on women in menopause. Korean J. For. Econ..

[22] Kim K.S. (2020). Influence of forest healing programs on health care of cancer patients—Mainly about physiological characteristics and psychological traits. J. Humanit. Soc. Sci..

[23] Lee M.M., Park B.J. (2020). Effects of forest healing program on depression, stress and cortisol changes of cancer patients. J. People Plants Environ..

[24] Bielinis E., Jaroszewska A., Łukowski A., Takayama N. (2020). The effects of a forest therapy programme on mental hospital patients with affective and psychotic disorders. Int. J. Environ. Res. Public Health.

[25] Jeon A.Y., Lee K.S., Lee S.M. (2019). Effects of the forest experience intervention program on depression, cognitive function, and quality of life in the elderly people with mild cognitive impairment. Korean J. Health Educ. Promot..

[26] Kim E.J., Park J.H., Sung K.M. (2017). The effectiveness of forest walking program on stress and recovery of schizophrenic patients in a closed ward. J. East-West Nurs. Res..

[27] Yeon P.S., Kim I.O., Kang S.N., Lee N.E., Kim G.Y., Min G.M., Chung C.Y., Lee J.S., Kim J.G., Shin W.S. (2022). Effects of urban forest therapy program on depression patients. Int. J. Environ. Res. Public Health.

[28] You J.H., Kim N.H., Sim H.R., Shin W.S. (2024). Concept and application of korean forest therapy prescriptions: An exploration of expert opinions through focus group interview. J. Korean Inst. For. Recreat..

[29] Korea Forest Welfare Institute https://www.fowi.or.kr.

[30] Kim S.J., Kim H.J., Lee K.J., Lee S.O. (2008). Focus Group Research Method.

[31] Krueger R.A., Casey M.A. (2014). Focus Groups: A Practical Guide for Applied Research.

[32] Stewart D.W., Shamdasani P.N. (2015). Focus Groups: Theory and Practice.

[33] Holsti O.R. (1969). Content Analysis for the Social Sciences and Humanities.

[34] Krippendorff K. (1989). Content analysis. Int. Encycl. Commun..

[35] Lal Das D.K., Bhaskaran V., Prasad B.D. (2008). Content analysis. Research methods for Social Work.

[36] Kim D., Lee J., Jeong I., Kim T., Choi M., Baek S. (2023). Development of a model of rehabilitation exercise and sports service delivery system for health promotion of people with disabilities. J. Exerc. Rehabil..

[37] Choi J.H., Kim H.J. (2020). Development of Forest-based customized health care platform and verification of service effectiveness. J. Korean Inst. For. Recreat..

[38] Shin W.S., Jeong J.Y., Kang S.N., Kim G.Y., Kim N.H., Kim J.G., Kim I.O., Min G.M., Sim H.L., Lee N.E. (2023). Urban Forest Face-to-Face/Non-Face-to-Face Forest Healing Program Guidebook for Depressed Patients.

[39] Kim H.S., Ko Y.J. (2022). Proposal of treatment system and outpatient treatment order system model using application non-face-to-face treatment and prescription: Focusing on domestic and case studies and feedback analysis. J. Reinterpretation Disabil..

[40] Doctor on Demand. https://doctorondemand.com.

[41] Kim S.K., Lee S., Kim T., Kim A., Jang Y., Lee S.H. (2020). Construction of prescription support system based on korean medicine ontology. J. Knowl. Inf. Technol. Syst..

[42] Jung Y., Im B., Kim H. (2010). A study on the role of hospital coordinator in primary health clinics. Korean J. Health Serv. Manag..

[43] Kim S.N. (2023). Literature on Job Analysis of medical institution coordinators: A systematic review. Korean J. Health Serv. Manag..

[44] Doty M.M., Fryer A., Audet A. (2012). The role of care coordinators in improving care coordination: The patient’s perspective. Arch. Intern. Med..

[45] Cho B.R., Cho M.H., Kim H.K., Park H.J., Jeong S.M. (2018). Development of Korean Care Coordinator and Care Model for Chronic Disease Management in Primary Care.

[46] Polley M.J., Fleming J., Anfilogoff T., Carpenter A. (2017). Making sense of social prescribing. Making Sense of Social Prescribing.

[47] Korea Forest Welfare Institute https://www.sooperang.or.kr.

[48] Lee D.S., Lee K.M., Lee H.J., Hwang J.H., Lee M.W., Cho S.H., Kim D.M., Woo H.B., Kwon O.S. (2021). A Research Report on Forest Healing Resources in 2021.

[49] Yoo J.H., Shin W.S., Kim N.H., Baek K.S., Shim H.R., Park S.Y. (2024). A Research Report on the Development of Health Promotion Effect Measurement Tools for Users of Forest Healing Services.

[50] Ha S.Y. (2024). Selection of an Index for Big Data of Forest Healing and Development of a Model Connecting to Health and Medical Service.

[51] Tsunetsugu Y., Park B.J., Miyazaki Y. (2010). Trends in Research Related to “Shinrin-yoku” (taking in the forest atmosphere or forest bathing) in Japan. Environ. Health Prev. Med..

[52] Li Q. (2013). What Is Forest Medicine? In Forest Medicine.

[53] Yi Y., Seo E., An J. (2022). Does Forest Therapy Have Physio-Psychological Benefits? A Systematic Review and Meta-Analysis of Randomized Controlled Trials. Int. J. Environ. Res. Public Health.

[54] Korea Forest Service https://map.forest.go.kr.

[55] Natural Resources Canada https://geo.ca.

[56] U.S. Department of Agriculture https://www.fs.usda.gov.

[57] Yoo R.H., Jeong M.A., Shin K.H. (2015). A study on the activation of forest therapy by the trend analysis related to naturopathy. J. Korean Inst. For. Recreat..

[58] Ji Y.H., Ha M.L., Kim H., Lee C.K. (2023). A Study on the Forest Healing Program. J. Agric. Life Sci..

[59] Kim J.L. (2020). Health Care Quality Assessment in the United States: A Case Study.

